# Diagnosis of prostate cancer by detection of minichromosome maintenance 5 protein in urine sediments

**DOI:** 10.1038/sj.bjc.6605785

**Published:** 2010-07-20

**Authors:** T J Dudderidge, J D Kelly, A Wollenschlaeger, O Okoturo, T Prevost, W Robson, H Y Leung, G H Williams, K Stoeber

**Affiliations:** 1 Department of Pathology and Cancer Institute, University College London, Rockefeller Building, 21 University Street, London, WC1E 6JJ, UK; 2 Department of Urology, Royal Marsden NHS Foundation Trust, Fulham Road, London, SW3 6JJ, UK; 3 Wolfson Institute for Biomedical Research, University College London, Cruciform Building, Gower Street, London, WC1E 6BT, UK; 4 Department of Public Health and Primary Care, Centre for Applied Medical Statistics, University of Cambridge, Institute of Public Health, Forvie Site, Robinson Way, Cambridge CB2 0SR, UK; 5 Urology Clinical Research Group, Newcastle upon Tyne Hospitals NHS Foundation Trust, Freeman Hospital, Newcastle upon Tyne, NE7 7DN, UK

**Keywords:** biomarker, DNA replication licensing, Mcm5, prostate cancer, PSA

## Abstract

**Background:**

The accuracy of prostate-specific antigen (PSA) testing in prostate cancer detection is constrained by low sensitivity and specificity. Dysregulated expression of minichromosome maintenance (Mcm) 2–7 proteins is an early event in epithelial multistep carcinogenesis and thus MCM proteins represent powerful cancer diagnostic markers. In this study we investigate Mcm5 as a urinary biomarker for prostate cancer detection.

**Methods:**

Urine was obtained from 88 men with prostate cancer and from two control groups negative for malignancy. A strictly normal cohort included 28 men with complete, normal investigations, no urinary calculi and serum PSA <2 ng ml^–1^. An expanded control cohort comprised 331 men with a benign final diagnosis, regardless of PSA level. Urine was collected before and after prostate massage in the cancer patient cohort. An immunofluorometric assay was used to measure Mcm5 levels in urine sediments.

**Results:**

The Mcm5 test detected prostate cancer with 82% sensitivity (confidence interval (CI)= 72–89%) and with a specificity ranging from 73 (CI=68–78%) to 93% (CI=76–99%). Prostate massage led to increased Mcm5 signals compared with pre-massage samples (median 3440 (interquartile range (IQR) 2280 to 5220) *vs* 2360 (IQR <1800 to 4360); *P*=0.009), and was associated with significantly increased diagnostic sensitivity (82 *vs* 60% *P*=0.012).

**Conclusions:**

Urinary Mcm5 detection seems to be a simple, accurate and noninvasive method for identifying patients with prostate cancer. Large-scale prospective trials are now required to evaluate this test in diagnosis and screening.

Prostate-specific antigen (PSA) testing for prostate cancer has shifted the focus of diagnostic strategies from the evaluation of symptomatic men to the screening of asymptomatic men years before the disease is clinically evident. Indeed, PSA is used for informal screening, case finding, diagnosis, prognosis, staging, monitoring of treatment and identification of recurrence (Oesterling, 1991). As a result, there has been an increase in the incidence of prostate cancer and the disease is now treated at an earlier stage (Hernandez and Thompson, 2004).

However, PSA testing is constrained by low sensitivity and specificity, reflected by its low area under the receiver operating characteristic (ROC) curve (AUC=0.678) (Thompson *et al*, 2005). Therefore, many men undergo potentially unnecessary diagnostic prostate biopsies that are painful and associated with risks of sepsis and bleeding. PSA testing inevitably leads to the overtreatment of some men, as the biopsy pathology, clinical findings and PSA level do not adequately predict prognosis. Indeed, it has been shown that preventing one prostate cancer-related death requires between 17 and 48 radical prostatectomies (Holmberg *et al*, 2002; Schroder *et al*, 2009). Thus, more accurate, noninvasive testing methods are needed to identify life-threatening but curable disease.

The initiation of DNA replication represents a final and critical step in growth control downstream of complex and redundant oncogenic growth signalling pathways, and is therefore a potentially attractive diagnostic and therapeutic target (Williams and Stoeber, 2007). Minichromosome maintenance (Mcm) proteins 2–7, the core components of the DNA replication initiation machinery, participate in the assembly of pre-replicative complexes (pre-RCs) on chromatin in G1 phase of the cell cycle, establishing competence for initiation of DNA synthesis in the subsequent S phase (Machida *et al*, 2005; Remus and Diffley, 2009). The six Mcm proteins (MCM) constitute the DNA replicative helicase and are therefore essential for chromosomal duplication. They are expressed throughout all phases of the cell division cycle, but are tightly downregulated in quiescent (G0), differentiated and senescent out-of-cycle states, and thus represent novel biomarkers of growth (Eward *et al*, 2004; Barkley *et al*, 2007; Williams and Stoeber, 2007). We and others have shown in a number of epithelial organ systems that MCMs become dysregulated and overexpressed in hyperproliferative dysplastic (preinvasive) and malignant states, resulting in exfoliation of MCM-positive tumour cells (Williams *et al*, 1998; Going *et al*, 2002; Williams and Stoeber, 2007). Moreover, the detection of exfoliated MCM-positive tumour cells in urine sediments, cervical Papanicolaou smears or gastroendoscopic-derived samples can be used in the detection of bladder, cervical, oesophageal, pancreatic and biliary tract cancers (Williams *et al*, 1998, 2004; Freeman *et al*, 1999; Stoeber *et al*, 2002; Ayaru *et al*, 2008).

We previously observed that Mcm5 is overexpressed in prostate cancer and that raised Mcm5 protein levels are an independent predictor of survival on multivariate analysis in patients treated with radical surgery (Meng *et al*, 2001) or androgen deprivation therapy and radiotherapy (Dudderidge *et al*, 2007). Moreover, we showed that elevated MCM expression in prostate cancer is coupled to arrested tumour differentiation and increased activation of the mitogen/extracellular-signal-regulated kinase kinase 5/extracellular signal-regulated kinase 5 (MEK/ERK5) pathway (Dudderidge *et al*, 2007). In contrast to premalignant and malignant states, MCMs are expressed at very low levels in benign prostatic tissue, with <2% of basal cells in normal and hyperplastic glands showing Mcm2 expression (Meng *et al*, 2001; Dudderidge *et al*, 2007). These findings suggest that Mcm 2–7 might be exploited as biomarkers for prostate cancer detection. Interestingly, in a study of Mcm5 as a biomarker for bladder cancer, 12 patients presenting with haematuria were identified with a new diagnosis of prostate cancer. These men had higher Mcm5 levels in urine sediments than men without malignancy (*P*<0.001). Notably, Mcm5 was undetectable in 70 patients with benign prostatic hyperplasia (Stoeber *et al*, 2002). Taken together, these studies raise the possibility that detection of elevated levels of Mcm5 may allow identification of prostate cancer patients with clinically significant tumours.

Urine, expressed prostatic secretions and semen could all provide suitable diagnostic material for biomarker detection of prostate cancer. Semen collection is awkward and not always possible, and the low volume of prostatic secretions restricts their use. The prostate cancer gene 3 (PCA3) test, a urinary diagnostic test for prostate cancer, uses a first-catch urine sample collected after a brief prostate massage (post-massage sample). The PCA3 assay identifies non-coding mRNA from the *PCA3* gene that is overexpressed in prostate cancer (Hessels and Schalken, 2009). The biological significance of this gene is unknown. However it seems to be a useful diagnostic tool with an AUC value for prostate cancer detection of 0.68 (Chun *et al*, 2009). Thus post-massage urine samples seem to be an effective target for prostate cancer detection.

In this pilot study we test the hypothesis that Mcm5 levels are increased in urine sediments from men with prostate cancer compared with men with no evidence of bladder or prostate cancer. We also measure the effect of prostate massage on Mcm5 levels.

## Materials and methods

### Study subjects

Patients attending urology or oncology clinics were recruited from Addenbrooke's Hospital (Cambridge, UK) and Freeman Hospital (Newcastle upon Tyne, UK). All patients gave written consent before being recruited to the study. Ethical approval was granted locally for each institution (Cambridge Local Research Ethics Committee reference no. 00/236, Newcastle Local Research Ethics Committee reference no. 02/161). All consecutive patients with a known diagnosis of prostate cancer who attended during the study period when researchers were present were recruited and categorised into pre- and post-treatment groups. Patients undergoing investigation for prostate cancer, and who subsequently had prostate cancer identified, were also recruited to the prostate cancer cohort. Patients who refused or could not give informed consent, who had undergone recent urological instrumentation or who had current/previous urothelial cancer were excluded. Where possible, samples were obtained from patients both before and after a brief prostate massage. Pre-massage samples comprised a whole voided urine specimen. Those patients who underwent prostate massage provided a second sample. Prostate massage involved 5–10 strokes lateral to medial on each prostatic lobe. Any prostatic secretions expressed per urethra were combined with a further whole voided urine specimen. Relevant clinical data, including serum PSA and urine NMP22 concentrations, Gleason grade and Gleason score, clinical stage, imaging determined lymph node status and bone scan status, were recorded prospectively from clinical notes and pathology records.

Men comprising the control groups were recruited from haematuria clinics and only later included in the control arm if investigations met strict selection criteria intended to exclude those with coexisting genitourinary tract malignancy. A stringently selected control group was identified to only include men with negative haematuria tests and with no past history of bladder or prostate cancer. Men with PSA levels >2 ng ml^–1^, NMP22 levels >7 U ml^–1^, abnormal or atypical urine cytology or incomplete haematuria investigations were excluded. Men with urolithiasis were also excluded because of the known effect of urothelial trauma causing release of MCM proteins from the dividing transit compartment of the epithelium (Stoeber *et al*, 2002; Williams and Stoeber, 2007). A second, expanded control group was identified to include all men with a benign final diagnosis and no past history of cancer, regardless of PSA measurements, that is, including patients for whom no PSA measurement was available. Patients with urinary calculi, incomplete investigations (e.g., visible haematuria but no contrast imaging study) and atypical cytology but with a benign final clinical diagnosis were not excluded from this group. To approximately age match the group with prostate cancer cases, men <50 years old were excluded. Patients who refused or could not give informed consent, who had undergone recent urological instrumentation or who had current or previous urothelial cancer were excluded. Urine was provided by men in these control groups without previous prostate massage.

### Processing of urine sediments

Urine sediments were obtained by centrifuging urine samples at 1500 **g** for 7 min at 4 °C. The pelleted material was resuspended in storage buffer (phosphate-buffered saline (PBS), 5% bovine serum albumin, 1 M sucrose and 0.02% NaN_3_) that contained one complete EDTA-free protease inhibitor cocktail tablet (Roche Diagnostics, Burgess Hill, UK) per 50 ml of buffer. The resuspended urine sediments were stored in liquid nitrogen within 2 h of the urine samples being passed.

### Preparation of standards for immunofluorometric Mcm5 assay

HeLa S3 cells (ATCC CCL-2.2) were cultured as exponentially growing monolayers in Dulbecco's modified Eagle medium (Invitrogen, Paisley, UK) supplemented with 10% fetal calf serum (Invitrogen), 100 U ml^–1^ penicillin and 0.1 mg ml^–1^ streptomycin in a 37 °C humidified incubator in the presence of 5% CO_2_. HeLa cells were harvested after trypsinisation and diluted with storage buffer to concentrations of 1500, 5000, 15 000, 50 000, and 150 000 cells per well. The zero-cell standard consisted of 500 *μ*l of storage buffer. The standards were stored in liquid nitrogen and later used to generate a standard curve for Mcm5.

### Processing of standards and clinical samples

Standards and clinical samples were thawed, and cells were isolated by centrifugation at 1500 **g** for 5 min at 4 °C. The supernatants were discarded, and cell pellets were washed three times with 500 *μ*l of PBS. Cell pellets were resuspended in 250 *μ*l (for those pellets with a volume approximately <200 *μ*l) or 500 *μ*l (for those pellets with a volume approximately >200 *μ*l) of processing buffer (PBS, 0.4% sodium dodecyl sulphate and 0.02% NaN_3_). Cell lysates were prepared by incubating the resuspended samples at 95 °C for 45 min. The DNA in each sample was sheared by passing the lysates through a 21-gauge needle (Terumo Europe, Leuven, Belgium), and nucleic acids were digested with DNase I (20 U ml^–1^; Roche Diagnostics) and RNase A (1 *μ*g ml^–1^; Roche Diagnostics) for 2 h at 37 °C. The samples were centrifuged at 15000 **g** for 10 min to pellet the cell debris, the supernatants were collected, and 50 *μ*l of each was directly used in the immunofluorometric assay.

### Immunofluorometric measurement of Mcm5 levels in urine sediments

Monoclonal antibodies (MAbs) 12A7 and 4B4 raised against His-tagged human Mcm5 were protein A-purified from hybridoma supernatants as described (Stoeber *et al*, 2002). Monoclonal antibody 4B4 was labelled with europium using a DELFIA Eu-labelling kit (PerkinElmer, Turku, Finland) according to the manufacturer's instructions. The assay was standardised using HeLa cells, and one fluorescence unit was defined as the signal generated by the Mcm5 contents of one proliferating HeLa cell, approximately 10^5^ Mcm5 molecules (Kearsey and Labib, 1998). DELFIA research reagents were obtained from PerkinElmer. All other reagents were obtained from Sigma-Aldrich (Dorset, UK). Multibuffer was prepared from 0.2 vol 5 × DELFIA assay buffer (PerkinElmer), 0.125 vol DELFIA TSH-Ultra assay buffer (PerkinElmer) and 0.1 vol Tween 20 (Sigma-Aldrich). Immunofluorometric measurements of Mcm5 levels were performed as described (Stoeber *et al*, 2002). Standard curves were constructed from fluorescence values generated by the blank and standard wells, and the fluorescence values of the urine sediment samples were calculated with the Multicalc Advanced Immunoassay Data Management package (PerkinElmer). For immunofluorometric measurement of Mcm5 levels, assay standards, control urine sediment samples and urine sediments from prostate cancer patients were run as duplicates and the mean of the duplicate results reported. For acceptance of immunofluorometric measurements in the assay, the following coefficients of variation were required: CV <20% for results between 1500 and 5000 cells per well standard curve points; CV <15% for results between 5000 and 15 000 cells per well; and CV <10% for results of >15 000 cells per well.

### Immunoassay performance

In our analysis of patient samples, we used 1800 cells per well as the lower detection limit because the within-batch variation of the assay for cell dilutions <1800 cells per well was >20%. Samples that generated an immunofluorometric signal below that corresponding to 1800 cells per well were reported as having <1800 cells per well.

### Statistical analysis

The amplitude of the Mcm5 fluorescence signal for each patient subgroup is presented as the median value with an associated interquartile range (IQR). Test performance was evaluated by calculating sensitivity and specificity using SPSS software, version 12.0.1 (SPSS, Chicago, IL, USA). An exact 95% confidence interval (CI) for each proportion was derived assuming a binomial distribution. Pre- and post-massage Mcm5 fluorescence signals were compared using the Wilcoxon signed-rank test for paired data. The difference in test performance for pre- and post-massage samples was assessed by comparing each sensitivity figure using McNemar's test. The *χ*^2^ tests were used to compare the sensitivity in different subgroups. All statistical tests were two tailed, and a 5% level was used to indicate statistical significance.

## Results

### Prostate cancer cohort demographics

Clinical characteristics, including age, serum PSA level, stage, grade distribution and treatment status for the cancer patient cohort, are shown in Table 1. In all, 88 men with prostate cancer were recruited with a median age of 72 years (IQR 66–77 years) and a median PSA concentration of 7.8 ng ml^–1^ (IQR 3.8–23.7 ng ml^–1^). The group was heterogeneous and included patients at all stages in the diagnostic and treatment pathway. The majority of patients were in clinical stage T1 (27%) and T2 (38%), with lower proportions in T3 (22%) and T4 (3%). Of the 88 prostate cancer patients, 39 were classified as ‘untreated’: 10 under active surveillance, 21 with newly diagnosed cancer attending for treatment decisions, 6 under investigation leading to a diagnosis of prostate cancer and 2 initially diagnosed with benign disease whose repeat biopsies showed prostate cancer. The median age of the untreated group was 69 years (IQR 65–73 years). The remaining ‘treated’ group included 49 patients with previous or ongoing treatment for prostate cancer, either androgen deprivation therapy, radiotherapy, chemotherapy or a combination of these treatments (Table 1). The median age of the treated patient cohort was 74 years (IQR 68–80 years), consistent with a more advanced stage of disease progression. Of the 49 treated patients, the majority were on luteinising hormone-releasing hormone (LHRH) analogues alone (31% including patients awaiting radiotherapy), maximum androgen blockade with LHRH analogues and anti-androgens (20%) or on LHRH analogues after previous treatment with radiotherapy (24%).

### Control cohort demographics

The clinical characteristics of both control cohorts are presented in Table 2. For the strictly normal control group, 135 men with PSA measurements and complete haematuria investigations were initially recruited. Patients with a past history or investigations suggesting bladder or prostate cancer, PSA concentration >2 ng ml^–1^, urine NMP22 concentration >7 U ml^–1^, urolithiasis or visible haematuria and incomplete imaging were excluded, leaving 28 men in the control cohort. This highly selected group had a median age of 60 years (IQR 54–68 years) and a median serum PSA concentration of 0.8 ng ml^–1^ (IQR 0.5–1.3 ng ml^–1^). The presenting complaints were asymptomatic nonvisible haematuria (*n*=8), symptomatic nonvisible haematuria (*n*=11), painless visible haematuria (*n*=6), painful visible haematuria (*n*=1) and haematospermia (*n*=2). Normal findings were identified in 11 men. Benign prostatic hyperplasia/obstruction was identified in seven men, prostatitis in five men and urinary tract infection in five men. Thus, although this group does not reflect a normal population, it does reflect typical patients attending urology clinics for investigation, and in whom malignancy can be excluded with a high degree of certainty. For the expanded normal control group, 755 men were recruited in total. In all, 331 men were identified from this group as potential controls after exclusion of men <50 years old with either a history of bladder or prostate cancer, cancer identified during investigation or a positive NMP22 result (>7 U ml^–1^). The median age of this group was 68 years (IQR 59–75 years). For 55 men in this expanded cohort, PSA measurements were available. The median value was 1.8 ng ml^–1^ (IQR 0.8–4.95 ng ml^–1^). The presenting complaints were asymptomatic nonvisible haematuria (*n*=75), symptomatic nonvisible haematuria (*n*=50), visible haematuria painless (*n*=124), visible haematuria painful (*n*=46), haematospermia (*n*=3) and unrecorded/other (*n*=33). Normal findings were identified in 154 men. Benign prostatic hyperplasia/obstruction was identified in 73, urinary calculi in 30, prostatitis in 7, urinary tract infection in 36, urethral stricture in 10, nephrological disease in 6 and other benign diagnoses in 15 men. This larger group contained men with no identifiable or previous cancer, but with the exception of a small group tested with PSA, no specific tests to exclude prostate cancer were made and thus a degree of contamination with occult tumours is to be expected.

### Urinary Mcm5 detection in prostate cancer patients and normal controls

The cohort of 88 prostate cancer patients recruited for study together provided 83 pre-massage and 60 post-massage urine samples for Mcm5 immunofluorometric analysis. Table 3 and Supplementary Figure 1 show the median Mcm5 signal for pre- and post-massage urine samples, for the highest Mcm5 signal group (i.e., Mcm5 signal for pre- or post-massage urine sample, whatever the higher), and for both control groups. The median for the highest Mcm5 signal group (3560 (IQR 2430–5575)) was significantly higher compared with the strictly defined controls (<1800 (IQR <1800 to <1800); *P*<0.001). Table 4 shows the sensitivity of cancer detection for pre- and post-massage samples, and for the highest Mcm5 signal group, as well as specificity values for the two control groups. In addition, 2 × 2 tables based on the highest recorded Mcm5 signal for all study participants and patients with pre-massage data only are shown in Supplementary Tables 1 and 2, respectively. As maximum sensitivity was desired, samples were scored as Mcm5 test positive if they generated a signal above the lower threshold limit for detection of Mcm5 protein in the immunoassay (1800 cut point). Using the maximum Mcm5 value obtained for each patient (i.e., either the pre- or post-massage Mcm5 signal), the overall sensitivity was 82% (72 out of 88; CI=72–89%) and specificity was 93% (26 out of 28; CI=76–99%). The sensitivity using the pre-massage samples only was 65% (54 out of 83; CI=54–75%) and the sensitivity using the post-massage samples only was 78% (47 out of 60; CI=66–88%). Of the 26 strictly normal control patients who tested negative, 7 were diagnosed with benign prostatic hyperplasia. In a previous study we reported similar low Mcm5 signals for 70 men with benign prostatic hyperplasia, indicating that the specificity of the Mcm5 test is unaffected by this common condition (Stoeber *et al*, 2002). Interestingly, both of the strictly normal control patients who had elevated Mcm5 signals and tested positive presented with haematospermia. To consolidate our finding that Mcm5 is of diagnostic utility in prostate cancer detection, further analysis was performed using less stringent exclusion criteria to identify an expanded control cohort. The median Mcm5 signal in this group was <1800 (IQR <1800 to 1950; Table 3) and was still significantly lower than the cancer group (*P*<0.001). Using this expanded control cohort, high specificity was still observed at 73% (242 out of 331; CI=68–78% Table 4 and Supplementary Figure 1). The false positives detected in this expanded control group (Mcm5 signal >1800) included normals without identifiable pathology (42 out of 154, 27%), urinary tract infection (10 out of 36, 28%) and urethral stricture (4 out of 10, 40%), benign prostatic hyperplasia (17 out of 73, 23%), calculi (15 out of 30, 50%), prostatitis (2 out of 7, 29%) and others (3 out of 15, 20%).

The effect of prostate massage on the Mcm5 signal and its effect on diagnostic sensitivity were also studied. From the prostate cancer cohort, 55 men provided both pre- and post-massage samples. As shown in Table 3 and Supplementary Figure 1, prostate massage led to a significant increase in median Mcm5 signal from 2360 (IQR <1800 to 4360) to 3440 (IQR 2280 to 5220; *P*=0.009). The increased amplitude of the Mcm5 signal after prostate massage was associated with a significant increase in diagnostic sensitivity from 60 (CI=46–73%) to 82% (CI=69–91% *P*=0.012; Table 4).

To determine the relationship between Mcm5 signal and PSA level, prostate cancer patients were grouped by PSA concentration: <5, 5–15 and >15 ng ml^–1^. All three groups showed elevated Mcm5 signals and similarly high sensitivities (Table 5 and Supplementary Figure 2). Notably, when considering the highest Mcm5 signal per patient, prostate cancer patients with a low PSA level (<5 ng ml^–1^; *n*=26) had a median Mcm5 signal of 3170 (IQR 2152–5887) and associated sensitivity of 81% (CI=61–93%), equivalent to prostate cancer patients with high PSA values (>15 ng ml^–1^; *n*=30) who had a median Mcm5 signal of 4065 (IQR 2450–5337) and associated sensitivity of 83% (CI=65–94% *P*=0.88).

To investigate the relationship between clinical characteristics and Mcm5 signal, the cancer patient cohort was further subclassified according to clinical stage (T1, T2 and T3/4), Gleason score (⩽6, 7 and 8–10), lymph node involvement and distant metastasis. The Mcm5 signal and sensitivity of the test were not influenced by any of these clinicopathological variables (Table 5, Supplementary Table 3 and Supplementary Figures 3 and 4). Surprisingly, the overall treatment status of patients did not seem to have a major effect on the Mcm5 signal. When the sensitivities for untreated (87% (CI=73–96%)) and treated patients (78% (CI=63–88%)) were compared, no statistical difference was observed between the two (*P*=0.36; Supplementary Table 4). However, considering only patients who provided a sample after prostate massage, we noted a decrease in median Mcm5 signal in those patients who underwent radiotherapy (2820 (IQR <1800 to 3960)) relative to those who did not undergo radiotherapy (3870 (IQR 2550 to 5870); Supplementary Table 4). This decrease in Mcm5 signal was associated with a significantly decreased sensitivity (54% (CI=25–81%) *vs* 87% (CI=70–96%); *P*=0.04).

## Discussion

There is an urgent need for more accurate, noninvasive diagnostic testing for prostate cancer. Although serum PSA-based screening has been shown to reduce prostate cancer mortality by 20%, there is a high risk of diagnosis of clinically insignificant tumours (Schroder *et al*, 2009). Recent data from the European Randomized Study of Screening for Prostate Cancer (ERSPC) showed that 1410 men would require screening and an additional 48 cancers treated to prevent one death from prostate cancer (Schroder *et al*, 2009). Moreover, the high PSA false-positive rate observed in this study (76%) shows the high number of prostate biopsies that might be avoided if a more accurate diagnostic test was available. Crucially, the disproportionate number of cancers that must be treated to prevent one death underlines the inadequate understanding of prostate cancer biology. Future diagnostic methods must not only reduce unnecessary prostate biopsies, but also provide prognostic information to aid treatment decisions. Moreover, the ideal diagnostic marker would preferentially identify clinically significant prostate cancers with a high risk of progression.

In this study we have identified Mcm5 as a potentially important biomarker for prostate cancer detection. Principally, we have shown that Mcm5 levels are elevated in the urine cell sediments of prostate cancer patients when compared with patients without malignancy. Only two patients had an elevated Mcm5 signal in the strictly normal control group. Interestingly, both patients presented with haematospermia and normal clinical investigations. This symptom is often because of prostatic calculi causing trauma to the prostatic ducts (Kumar *et al*, 2006). This would potentially expose the normal MCM-expressing proliferative compartment to the lumen of these ducts, and may thus explain the two false-positive results. Similarly, patients in the expanded haematuria control group diagnosed with bladder calculi also showed elevated Mcm5 signals. This confirms our previous findings that calculi in the genitourinary tract generate elevated Mcm5 signals in urine cell sediments (Stoeber *et al*, 2002). Importantly, the 73 patients with BPH included in this study did not show elevated Mcm5 signals compared with those controls without pathology. Similarly, 70 patients with BPH analysed as part of a previous Mcm5 bladder cancer trial did not show elevated Mcm5 levels (Stoeber *et al*, 2002). This is consistent with the fact that MCM expression levels in hyperplastic glands are very low (<2% of basal cells), similar to those observed in normal prostatic tissue (Meng *et al*, 2001; Dudderidge *et al*, 2007). Notably, prostatitis was associated with an elevated Mcm5 signal.

A reduction in specificity of the Mcm5 test, from 93 to 73%, was observed when using the expanded control cohort. Although this measure of specificity was derived from a much larger population, the PSA values were unknown for the majority. This expanded control cohort is therefore likely to include occult prostate cancers. Indeed, if one considers that the prevalence of prostate cancer in the Prostate Cancer Prevention Trial was 20.7% in the 55- to 59-year-old group (in a group originally comprising patients with normal digital rectal examination and PSA <3 ng ml^–1^; Thompson *et al*, 2003), one might expect a significant number of occult prostate cancers to be included within our expanded control population. Interestingly, the Mcm5 false-positive rate in the normal population without detectable pathology was 27%. It will therefore be of interest in future studies to determine what percentage of these cases might represent occult prostate cancers.

In the context of prostate cancer detection, we have also established the importance of prostate massage in the preparation of urine samples for Mcm5 testing. This brief procedure is well tolerated by patients in the urology clinic and is an integral part of their clinical assessment. Little variation from standard examination is required to optimise sample quality. We have shown that massage not only increased Mcm5 signals in urine sediments, but it also led to significantly increased diagnostic sensitivity. These findings require verification in a larger study with all patients undergoing pre- and post-massage testing.

It is interesting to note that there was no major reduction in Mcm5 signals after medical therapeutic intervention. We have previously shown in normal tissues and tumours that cells arrested ‘in cycle’ can maintain MCM protein expression, designated as ‘licensed’ cells with proliferative potential (Stoeber *et al*, 2001; Dudderidge *et al*, 2005; Williams and Stoeber, 2007). Persistence of elevated Mcm5 signals in the urine sediments of treated patients suggests that there has been no significant reduction in tumour cell volume, and this may contribute to relapse after such medical therapeutic interventions. Disease progression could be triggered by the overriding of DNA damage cell cycle checkpoints or, alternatively, through the establishment of growth-independent (autonomous) cancer cell cycles.

Prostate-specific antigen is primarily an organ-specific marker and its elevation in prostate cancer is because of the leakage of a physiological protein into the blood, resulting from the disruption of the basement membrane (Lilja *et al*, 2008). Thus, PSA is not an ideal tumour marker as it is also elevated in other prostatic diseases that cause increased permeability of the basement membrane, including BPH and prostatitis (Lilja *et al*, 2008). In contrast, Mcm5 detection can be regarded as a cancer-specific test. Therefore, the combination of a cancer-specific test in Mcm5 with a prostate-specific test in PSA may provide an improved algorithm for prostate cancer detection.

Although this pilot study has identified Mcm5 as a new biomarker for prostate cancer detection, it is not yet clear whether the test will be able to specifically identify clinically significant cancers. Comparable sensitivities and Mcm5 signals were observed between low-grade, low-stage and high-grade, advanced-stage tumours. However, this is a heterogeneous patient cohort, in which many patients have undergone therapeutic intervention. Trials on a conventional diagnostic untreated patient cohort will be required to address this question. Future studies should also evaluate the combination and comparison of serum PSA, urinary PCA3 and Mcm5, and assess the use of a combined predictive biomarker algorithm that may allow identification of patients in whom prostate biopsy can be safely avoided.

## Figures and Tables

**Table 1:** Clinical characteristics of prostate cancer patients

**Characteristic**	***n* (%) or median (IQR)**
All patients	88
Age, years	72 (66–77)
PSA, ng ml^–1^	7.8 (3.8–23.7)^a^
	
*Clinical T stage*	
T1	24 (27)
T2	33 (38)
T3	19 (22)
T4	3 (3)
Tx	9 (10)
	
*Clinical M stage*	
M0	36 (41)
M1	18 (20)
Mx	34 (39)
	
*Clinical N stage*	
N0	38 (43)
N1	0 (0)
Nx	50 (57)
	
*Gleason score*	
⩽6	32 (36)
7	26 (30)
8	12 (14)
9	5 (6)
10	3 (3)
Unknown	10 (11)
	
*Treatment status*	
Before diagnosis	2 (2)
Untreated	37 (42)
Treated	49 (56)
LHRH only	15 (31)^b^
Antiandrogens alone	0 (0)
Radiotherapy alone	5 (10)
Chemotherapy alone	1 (2)
LHRH + antiandrogens	10 (20)
LHRH + radiotherapy	12 (24)
Radiotherapy + antiandrogens	1 (2)
Radiotherapy + chemotherapy	1 (2)
LHRH + radiotherapy + antiandrogens	3 (6)
LHRH + radiotherapy + chemotherapy	1 (2)

Abbreviations: LHRH=luteinising hormone-releasing hormone; PSA=prostate-specific antigen; IQR=interquartile range.

a *N*=85.

b Percentage of treated group (*n*=49); rounded averages add up to <100%.

**Table 2:** Clinical characteristics of cancer-free control patients

	***n* (%) or median (IQR)**	
**Characteristic**	**Strictly normal**	**Expanded control**
All patients	28	331
Age, years	60 (54–68)	68 (59–75)
PSA, ng ml^–1^	0.8 (0.5–1.3)	1.8 (0.8–4.95)^a^
		
*Initial referral*		
Asymptomatic nonvisible haematuria	8 (29)	75 (23)
Haematospermia	2 (7)	3 (1)
Indwelling catheter (haematuria)	0 (0)	1 (<1)
Symptomatic nonvisible haematuria	11 (39)	50 (15)
Visible haematuria, painful	1 (4)	46 (14)
Visible haematuria, painless	6 (21)	124 (37)
Unrecorded	0 (0)	32 (10)^b^
		
*Diagnosis on final evaluation*		
Normal	11 (39)	154 (47)^b^
Benign prostatic hyperplasia/obstruction	7 (25)	73 (22)
Nephrological	0 (0)	6 (2)
Prostatitis	5 (18)	7 (2)
Urinary calculi	0 (0)	30 (9)
Urethral stricture	0 (0)	10 (3)
Urinary tract infection	5 (18)	36 (11)
Other	0 (0)	15 (5)

Abbreviations: PSA=prostate-specific antigen; IQR=interquartile range.

a *N*=55, includes strictly normal patients and those with PSA levels >2 ng ml^–1^.

b Rounded percentages do not sum to 100%.

**Table 3:** Mcm5 signal for normal controls and prostate cancer patients

	***n***	**Mcm5, median (IQR)**
*All cancer patients*		
Before massage	83	2680 (<1800–4720)
After massage	60	3415 (2140–5190)
Highest Mcm5 signal	88	3560 (2430–5575)
		
*Cancer patients before and after massage*		
Before massage	55	2360 (<1800–4360)
After massage	55	3440 (2280–5220)^a^
Normal controls		
Strictly normal group – before massage	28	<1800 (<1800–<1800)
Expanded cohort – before massage	331	<1800 (<1800–1950)

Abbreviations: PSA=prostate-specific antigen; IQR=interquartile range; Mcm=minichromosome maintenance.

a Compared with before massage, *P*=0.009 (Wilcoxon's signed-rank test).

**Table 4:** Sensitivity and specificity analysis of Mcm5 test performance in normal controls and prostate cancer patients

	***n***		**% (CI)**
*All cancer patients*			
Before massage	83	Sensitivity	65 (54–75)
After massage	60	Sensitivity	78 (66–88)
Highest Mcm5 signal	88	Sensitivity	82 (72–89)
			
*Cancer patients before and after massage*			
Before massage	55	Sensitivity	60 (46–73)
After massage	55	Sensitivity	82 (69–91)^a^
Normal controls			
Strictly normal group – before massage	28	Specificity	93 (76–99)
Expanded cohort – before massage	331	Specificity	73 (68–78)

Abbreviations: CI=confidence interval; Mcm=minichromosome maintenance.

a Compared with before massage, *P*=0.012 (McNemar's test).

Sensitivity and specificity were determined using a cut point of 1800 for Mcm5 signal.

**Table 5:** Mcm5 signal test sensitivity in cancer patients categorized by PSA level, clinical stage and Gleason score

	**Pre-massage**						**Post-massage**					**Highest Mcm5 signal**			
	***n***	**Mcm5, median (IQR)**	**Sensitivity % (CI)**	*P* (*n*)*	***P* (*t*)^†^**	***n***	**Mcm5, median (IQR)**	**Sensitivity % (CI)**	***P* (*n*)**	***P* (*t*)**	***n***	**Mcm5, median (IQR)**	**Sensitivity % (CI)**	***P* (*n*)**	***P* (*t*)**
*PSA, ng* *ml*^–1^															
<5	25	2180 (<1800 to 4190)	56 (34–76)	—	—	20	3170 (1874 to 5587)	75 (51–91)	—	—	26	3170 (2152 to 5887)	81 (61–93)	—	—
5–15	26	2365 (<1800 to 3947)	62 (41–80)	0.77	0.21	23	3870 (2280 to 5160)	83 (61–95)	0.65	0.96	29	3870 (2250 to 5240)	79 (60–92)	0.95	0.80
>15	29	3440 (<1800 to 5075)	72 (53–87)	0.24	—	16	3290 (1914 to5037)	75 (48–93)	1.00	—	30	4065 (2450 to 5337)	83 (65–94)	0.88	—
															
*Clinical stage*															
T1	23	2460 (<1800 to 5000)	61 (39–80)	—	—	18	4485 (3210 to 7797)	94 (73–100)	—	—	24	4505 (3007 to 6900)	92 (73–99)	—	—
T2	30	2210 (<1800 to 3140)	60 (41–77)	0.98	0.70	28	2625 (<1800 to 4240)	68 (48–84)	0.08	0.06	33	2680 (2045 to 4445)	79 (61–91)	0.33	0.05
T3/4	21	3470 (<1800 to 5290)	67 (43–85)	0.75	—	9	2560 (<1800 to 4300)	67 (30–93)	0.22	-	22	3500 (<1800 to 5330)	68 (45–86)	0.10	—
															
*Gleason score*															
⩽6	32	2310 (<1800 to 3420)	62 (44–79)	—	—	24	3800 (2887 to5707)	87 (68–97)	—	—	32	3545 (2715 to 5707)	91 (75–98)	—	—
7	22	2125 (<1800 to 3580)	55 (32–76)	0.58	0.77	19	3660 (<1800 to 5160)	74 (49–91)	0.37	0.05	26	3375 (<1800 to 4830)	69 (48–86)	0.10	0.12
8–10	19	3530 (<1800 to 5660)	68 (43–87)	0.70	—	12	2340 (<1800 to 3212)	58 (28–85)	0.13	—	20	3255 (1942 to 5552)	75 (51–91)	0.27	—

Abbreviations: CI=confidence interval; Mcm=minichromosome maintenance; IQR=interquartile range; PSA=prostate-specific antigen.

^*^P-value for sensitivity, *vs* base category (*χ*^2^ test).

^†^*P-*value for trend across category.
