# Supplementary figures and images for: Diagnosis of prostate cancer by detection of minichromosome maintenance 5 protein in urine sediments
Source: Br J Cancer. 2010 Jul 20;103(5):701–7. doi: 10.1038/sj.bjc.6605785 (PMC2938246; doi:10.1038/sj.bjc.6605785)

Supplementary Figure 1

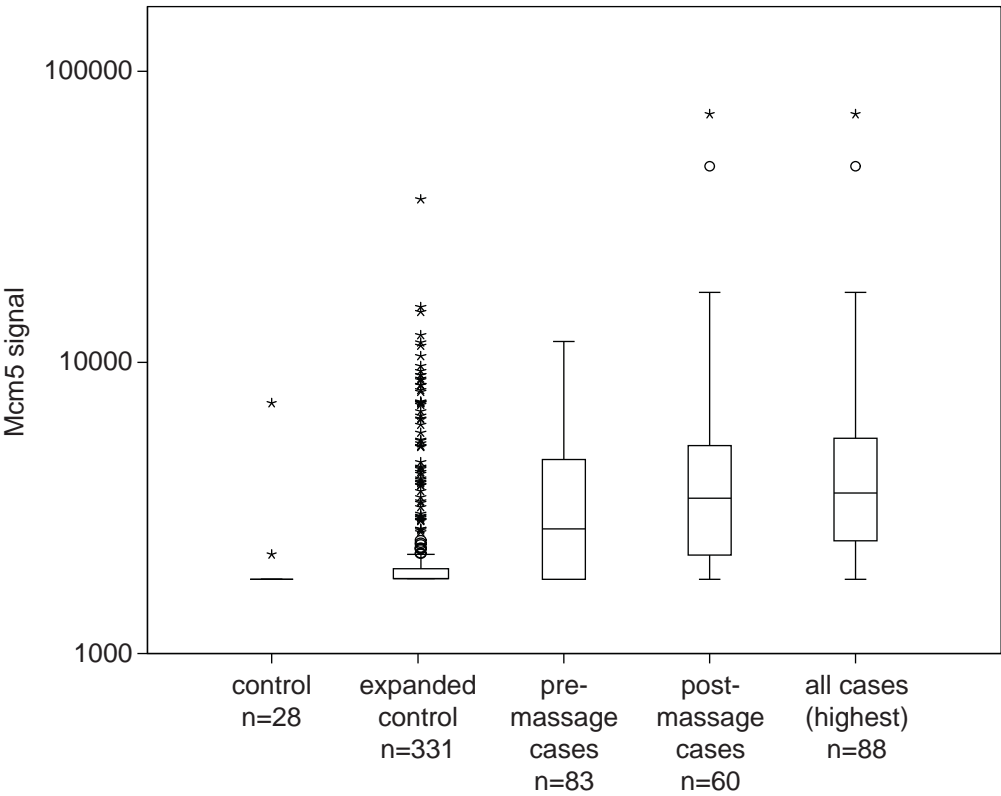

Supplementary Figure 2

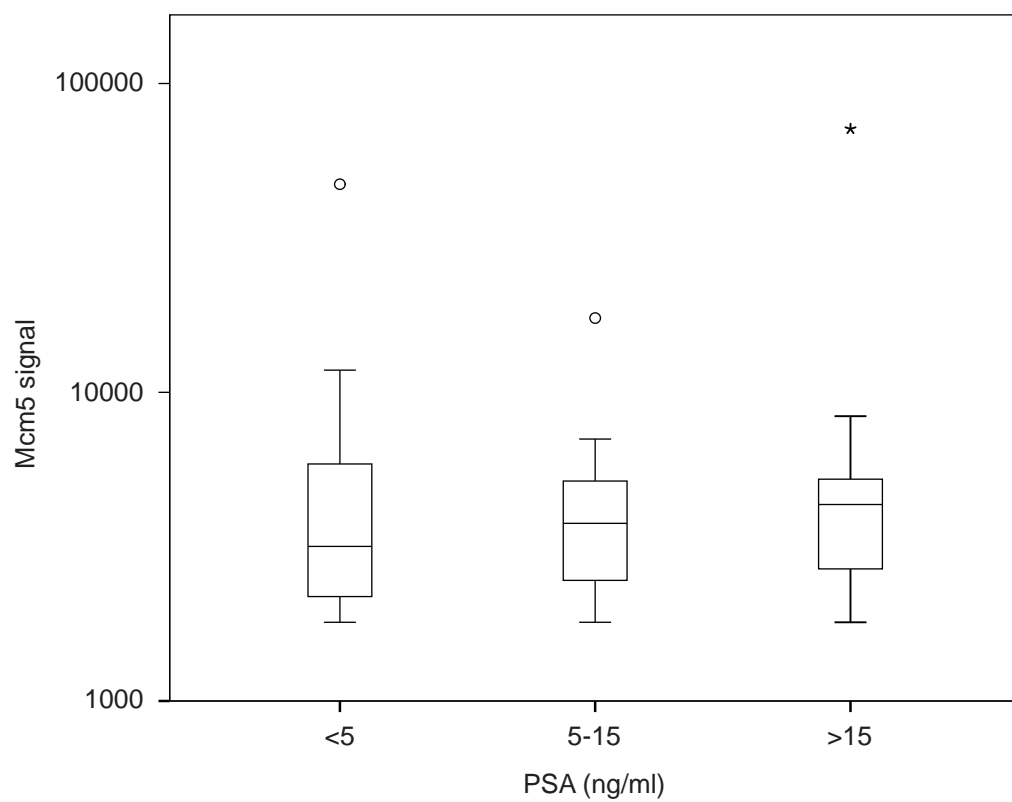

Supplementary Figure 3

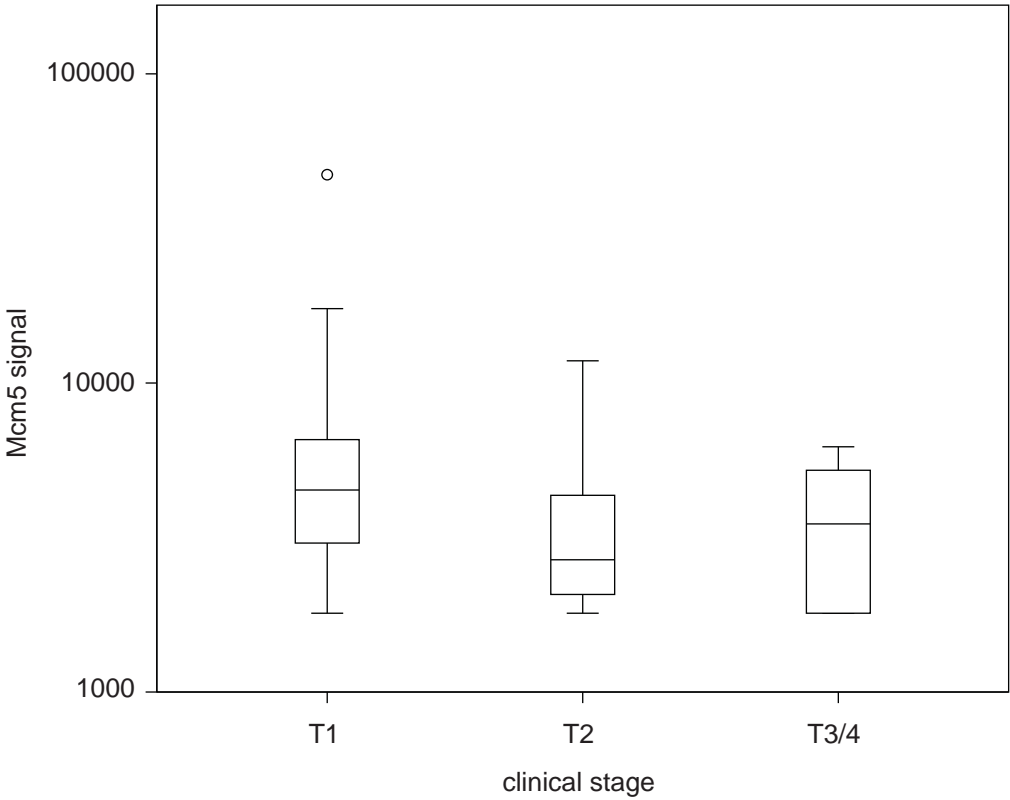

Supplementary Figure 4

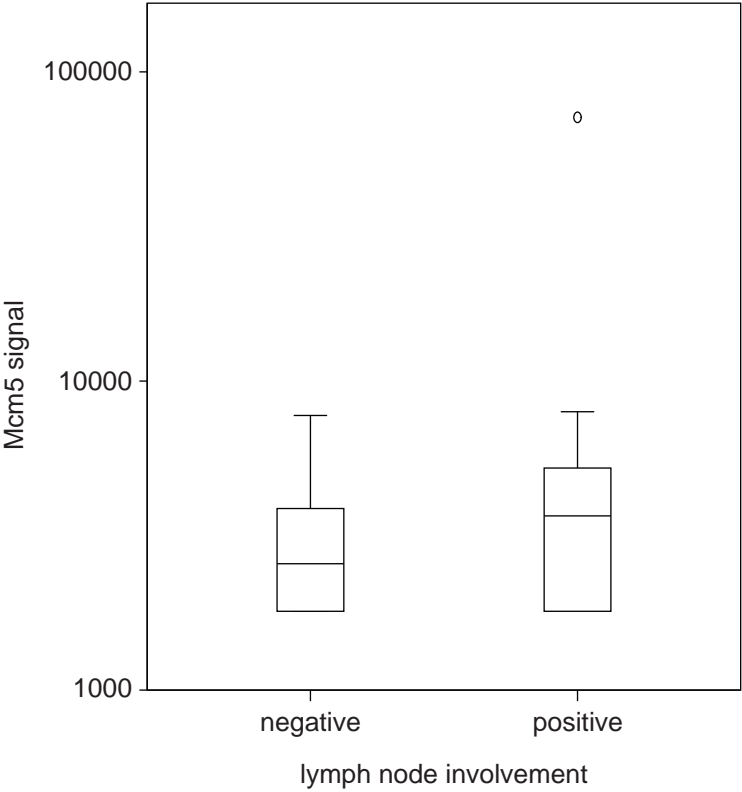

Supplement: Supplementary Figures 1–4 [file 6605785x1.pdf]
