# Supplementary material for: Diagnosis of prostate cancer by detection of minichromosome maintenance 5 protein in urine sediments
Source: Br J Cancer. 2010 Jul 20;103(5):701–7. doi: 10.1038/sj.bjc.6605785 (PMC2938246; doi:10.1038/sj.bjc.6605785)
Supplement: Supplementary Tables 1–4 [file 6605785x2.pdf]

Supplementary Table 1: 2x2 table for all study participants

	**Control**	**Case**	**Total**
Negative	26	16	42
Positive	2	72	74
Total	28	88	116

Patients determined to be positive for disease if highest Mcm5 signal available was above cut-point of 1800

[a] Chi-squared test P<0.001

Supplementary Table 2: 2x2 table for study participants with pre-massage data

	Control	Case	Total
Negative	26	29	55
Positive	2	54	56
Total	28	83	111

Patients determined to be positive for disease if highest Mcm5 signal available was above cut-point of 1800

[a] Chi-squared test P<0.001

Supplementary Table 3: Mcm5 signal and test sensitivity for cancer patients, categorized by bone scan and lymph node status

	Pre-massage				Post-massage				Highest Mcm5 signal			
	**n**	**Mcm5, median (IQR)**	**Sensitivity, % (CI)**	**P***	**n**	**Mcm5, median (IQR)**	**Sensitivity, % (CI)**	**P**	**n**	**Mcm5, median (IQR)**	**Sensitivity, % (CI)**	**P**
Bone scan												
Negative	32	2125 (<1800–3807)	56 (38–74)	–	26	2885 (<1800–4277)	62 (41–80)	–	36	3035 (<1800–4502)	69 (52–84)	–
Positive	18	4280 (1965–5960)	78 (52–94)	0.15	10	4660 (2492–8015)	90 (55–100)	0.13	18	5270 (3290–7795)	89 (65–99)	0.21
Lymph node												
Negative	23	2180 (<1800–3500)	57 (34–77)	–	13	2570 (<1800–4515)	69 (39–91)	–	26	2565 (<1800–4132)	65 (44–83)	–
Positive	10	3475 (<1800–4585)	60 (26–88)	0.91	5	2260 (<1800–37780)	60 (15–95)	0.78	10	3665 (<1800–5912)	70 (35–93)	0.85

* P value for sensitivity, versus base category (Chi-squared test)

Supplementary Table 4: Mcm5 signal and test sensitivity for cancer patients, categorized by treatment and radiotherapy status

	**Pre-massage**				**Post-massage**				**Highest Mcm5 signal**			
	**n**	**Mcm5, median (IQR)**	**Sensitivity, % (CI)**	**P***	**n**	**Mcm5, median (IQR)**	**Sensitivity, % (CI)**	**P**	**n**	**Mcm5, median (IQR)**	**Sensitivity, % (CI)**	**P**
Treatment status												
Untreated	37	2370 (<1800–3985)	57 (39–73)	–	31	3870 (2550–5870)	87 (70–96)	–	39	3870 (2550–5200)	87 (73–96)	–
Treated	46	3300 (<1800–5405)	72 (57–84)	0.15	29	3090 (<1800–4660)	69 (49–85)	0.15	49	3500 (2030–5870)	78 (63–88)	0.36
Radiotherapy												
No	37	2370 (<1800–3985)	57 (39–73)	–	31	3870 (2550–5870)	87 (70–96)	–	39	3870 (2550–5200)	87 (73–96)	–
Yes	21	2040 (<1800–5770)	57 (34–78)	1.00	13	2820 (<1800–3960)	54 (25–81)	0.04	23	3090 (<1800–5660)	70 (47–87)	0.16

* P value for sensitivity, versus base category (Chi-squared test)
