# Supplementary material for: Diagnosis of prostate cancer by detection of minichromosome maintenance 5 protein in urine sediments
Source: Br J Cancer. 2010 Jul 20;103(5):701–7. doi: 10.1038/sj.bjc.6605785 (PMC2938246; doi:10.1038/sj.bjc.6605785)
Supplement: Supplementary Figures 1–4 Legends [file 6605785x3.doc]

**Supplementary Data**

**Supplementary Figure Legends**

**Supplementary Figure 1: Box-whisker plots showing Mcm5 immunofluorometric signals grouped by control and patient cohort**. The data show the distribution of Mcm5 signals in the control and expanded control groups, and in the pre-massage, post-massage and highest Mcm5 signal groups. The median (line), interquartile range (boxed), and range (enclosed by lines) of Mcm5 signals are shown. Extreme outlying cases (stars) and outlying cases (circles) are depicted by isolated points.

**Supplementary Figure 2: Box-whisker plots of Mcm5 immunofluorometric signals grouped by serum PSA concentration**. The median (line), interquartile range (boxed), and range (enclosed by lines) of Mcm5 signals grouped by PSA level (<5, 5-15 and >5 ng/ml) are shown for study participants with valid PSA and Mcm5 measurements. Extreme outlying cases (star) and outlying cases (circles) are depicted by isolated points.

**Supplementary Figure 3: Box-whisker plots of Mcm5 immunofluorometric signals grouped by clinical stage.** The median (line), interquartile range (boxed), and range (enclosed by lines) of Mcm5 signals are shown for patients diagnosed with stage T1, T2 and T3/4 tumours. Outlying cases are depicted by isolated points.

**Supplementary Figure 4: Box-whisker plot of Mcm5 immunofluorometric signals grouped by lymph node status.** The median (line), interquartile range (boxed), and range (enclosed by lines) of Mcm5 signals grouped by lymph node involvement are shown. Outlying cases are depicted by isolated points.
